# Hybrid Network Structure of Hexagonal Boron Nitride-Silicon Carbide Whisker to Improve the Performance of the Polybenzoxazine with KH560-Boron Nitride

**DOI:** 10.3390/polym18070837

**Published:** 2026-03-29

**Authors:** Qi An, Kai Chong, Yaran Pei, Dengxia Wang, Jiakai Li, Keyong Xie, Xinbo Wang, Jingjing Liu, Siying Wang, Hui Li, Yan Sun

**Affiliations:** 1 Shandong Institute of Non-Metallic Materials, Jinan 250031, China; 2 Institute of Optical Physics and Engineering Technology, Qilu Zhongke, Jinan 250100, China

**Keywords:** polybenzoxazine, hybrid fillers, BN-MgO-SiC_w_, thermal conductivity, tensile strength, flexural strength

## Abstract

In this study, NH_2_-MgO was employed as a crosslinking agent to covalently link boron nitride (BN) and silicon carbide whiskers (SiC_w_) via an amidation reaction, yielding the BN-MgO-SiC_w_ hybrid filler. The BN-MgO-SiC_w_/PBz composites were fabricated using a ball-milling-assisted solution mixing method combined with hot-press molding, and their comprehensive properties were systematically evaluated. The results demonstrate that the BN-MgO-SiC_w_/PBz composite exhibits excellent thermal conductivity, favorable dielectric properties, superior thermal stability, and outstanding mechanical performance. At a filler loading of 50 wt%, the composite achieved a thermal conductivity of 1.41 W/mK, which is substantially higher than that of the KH560-BN/PBz composite (0.91 W/mK) and approximately 5.2 times that of the neat PBz matrix. The dielectric constant (ε) and dielectric loss (tan δ) of the BN-MgO-SiC_w_/PBz composite were 6.81 and 0.013, respectively, remaining at relatively low levels. The thermal degradation temperature at 30% weight loss (T_30_) and the heat resistance index temperature (T_HRI_) reached 572 °C and 244 °C, respectively, both higher than those of the KH560-BN/PBz composite at the same filler loading (511 °C and 224 °C). The tensile strength and flexural strength of the BN-MgO-SiC_w_/PBz composite were 50.0 MPa and 72.3 MPa, respectively, exceeding those of the KH560-BN/PBz composite (39.4 MPa and 56.2 MPa) while remaining slightly below those of the neat PBz matrix. Collectively, these findings indicate that the BN-MgO-SiC_w_/PBz composite holds great promise as a novel material with well-balanced comprehensive properties, making it a strong candidate for applications in fields such as electronic packaging.

## 1. Introduction

Polybenzoxazine (PBz) is a new type of phenol-formaldehyde resin (PF). Compared with conventional resins such as polyimide (PI), epoxy resin (EP), bismaleimide resin (BMI), PBz has more ideal thermal resistance performance and electrical insulation performance, which makes it widely used in the fields of electronics, aerospace [1,2,3,4]. However, the low thermal conductivity (0.2531 W/mK) of PBz limits its application in the field of microelectronic packaging [5,6,7]. Due to the rapid rise of integrated technology in the field of microelectronics, modern electronic equipment is constantly developing towards the direction of miniaturization and power density increase, resulting in microelectronics releasing a lot of heat during operation, which affects the accuracy and service life of the microelectronics equipment [8,9]. In order to effectively improve the heat transfer capability of electronic chips, research in recent years has focused on the preparation of ceramic/polymer composites with high thermal conductivity and electrical insulation properties [10,11,12,13,14,15]. Among various ceramic fillers, boron nitride (BN) is considered to be the ideal thermal conductive ceramic filler because of its excellent thermal conductivity, good electrical insulation performance and outstanding thermal stability [16,17,18,19,20].

In addition, establishing a good heat conduction network is an effective method to improve the comprehensive performance of polymer matrix composites [21,22]. Therefore, in order to achieve the synergistic effect of hybrid fillers, recent research has been dedicated to preparing high-performance polymer composites by adding composite fillers. For instance, Won et al. [23] added BN-SiC composite fillers to polymers through hydroxylation reactions, effectively enhancing the thermal conductivity of polymer composites. When the filling amount of the filler is 70 vol%, the thermal conductivity of the composite material is 1.61 W/mK. It is well known that the filling of a large number of heat-conducting particles can obtain composite materials with high thermal conductivity, but at the same time, it will inevitably lead to the deterioration of the mechanical properties of the composite materials. However, silicon carbide whiskers are single-crystal fibers with a certain aspect ratio, and thus are regarded as an ideal bridge material for connecting boron nitride [23,24,25,26]. Silicon carbide whiskers, as a high-performance ceramic filler, not only possess a high thermal conductivity but also exhibit excellent mechanical properties, which can significantly improve the poor mechanical performance of composite materials under high filler content [27,28]. In addition, the network structure formed between boron nitride and silicon carbide whiskers can construct the transmission path of phonons and electrons, which can effectively improve the thermal conductivity and electrical insulation performance of the composite material [29,30,31,32]. However, since both boron nitride and silicon carbide whiskers are chemically inert, it is difficult to form covalent connections. Therefore, the phase difference that can cause interfacial resistance exists between the whiskers of boron nitride and silicon carbide, hindering the migration of electrons and phonons and affecting the comprehensive performance of the composite material.

In this study, amino-modified magnesium oxide (NH_2_-MgO) was employed as a crosslinking agent to establish covalent connections between boron nitride (BN) and silicon carbide whiskers (SiC_w_) via an amidation reaction. Specifically, BN and SiC_w_ were first functionalized with γ-glycidoxypropyltrimethoxysilane (KH560), while MgO was functionalized with 3-aminopropyltrimethoxysilane (KH540). The resulting BN-MgO-SiC_w_/PBz composites were fabricated using a hot-pressing method. The covalently bonded network architecture formed by BN-MgO-SiC_w_ within the polymer matrix effectively reduces the interfacial thermal resistance between BN and SiC_w_, thereby facilitating enhanced electron and phonon transport.

Compared with previously reported systems, the BN-MgO-SiC_w_/PBz composites developed in this work exhibit comprehensive performance that is either superior to or comparable with similar material systems documented in the literature (Table 1). These results demonstrate that the BN-MgO-SiC_w_ hybrid structure, achieved through covalent linkage via NH_2_-MgO, significantly enhances the thermal conductivity, electrical insulation, and mechanical properties of the PBz-based composites.

## 2. Materials and Methods

### 2.1. Materials

Polybenzoxazine (PBz) was purchased from Shanghai Ruiyi Chemical Technology Co, Ltd. (Shanghai, China). Boron nitride (4–6 μm) was provided by Shandong Yasai Ceramic Technology Co., Ltd. (Jinan, China). Silicon carbide whiskers were received from Qinhuangdao Yinuo High-tech Material Development Co., Ltd. (Qinhuangdao, China). Spherical magnesium oxide was provided by Bohuasi Nano Technology Co., Ltd. (Ningbo, China). Silane coupler (KH560), 3-aminopropyltrimethoxysilane (KH540) and tetrahydrofuran (THF) were purchased from Shanghai Macklin Biochemical Technology Co., Ltd. (Shanghai, China).

### 2.2. Characterizations

Tensile strength and flexural strength were measured using a universal material testing machine (RGT-10A, Shenzhen Ruigeer Instrument Co., Ltd., Shenzhen, China) in accordance with GB/T 23805-2009. Microstructure characterization was performed using a scanning electron microscope (GEMINI 300, ZEISS, Jena, Germany). Fourier transform infrared (FTIR) spectra were recorded using a Fourier transform infrared spectrometer (SPECTRUM-400, PerkinElmer, Waltham, MA, USA), and Raman spectra were obtained using a Raman spectrometer (DXR SmartRaman, Thermo Fisher Scientific, Waltham, MA, USA). The thermal conductivity of the sample was detected by using a thermal conductivity tester with the direction of through-plane (GHP 456 model, NETZSCH GERAETEBAU GMBH, Selb, Germany). The dielectric constant (ε) and dielectric loss (tan δ) of the samples were measured using an E4980AL precision LCR meter (Keysight Technologies, Santa Rosa, CA, USA) at a frequency of 1 MHz. Thermogravimetric analysis (TGA) and derivative thermogravimetric (DTG) curves of the composites were obtained using a thermogravimetric analyzer (Netzsch, Selb, Germany) under a nitrogen atmosphere at a heating rate of 10 °C/min.

### 2.3. Synthesis of BN-MgO-SiC_w_ Hybrid Fillers

**Figure 1:**
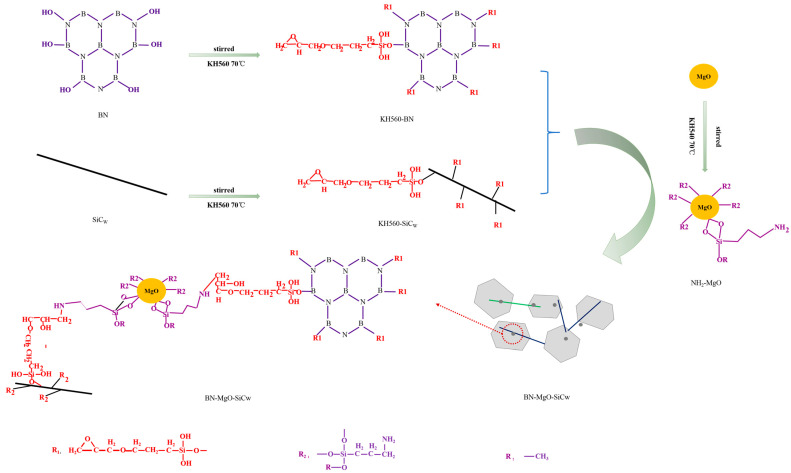
Synthesis process of BN-MgO-SiC_w_ hybrid fillers.

Figure 1 illustrates the synthesis process of the BN-MgO-SiC_w_ hybrid fillers. First, 1 g of KH560 and 20 g of BN were added to a mixed solution of ethanol and deionized water (250 mL:250 mL) and stirred at 70 °C for 6 h. The resulting powder was then repeatedly rinsed with deionized water to remove any residual KH560, and finally dried in vacuo at 60 °C for 8 h to obtain KH560-functionalized BN (KH560-BN). Second, the same procedure was applied to modify SiC_w_, yielding KH560-functionalized SiC_w_ (KH560-SiC_w_). Third, 1 g of MgO and 40 mL of KH540 were dissolved in 450 mL of ethanol. The mixture was sonicated for 1 h and then stirred at 60 °C for 10 h. Subsequently, the mixture was diluted with 1000 mL of ethanol solution, and the resulting NH_2_-functionalized MgO (NH_2_-MgO) was dried at 60 °C for 24 h. Fourth, 4 g of KH560-SiC_w_, 16 g of KH560-BN, and 0.04 g of NH_2_-MgO were added to 500 mL of tetrahydrofuran (THF), stirred for 0.5 h, and then sonicated for 1 h. The mixed solution was reacted at 60 °C for 4 h, and finally dried at 80 °C for 12 h to obtain the BN-MgO-SiC_w_ hybrid filler.

### 2.4. Preparation of PBz Composites

**Figure 2:**
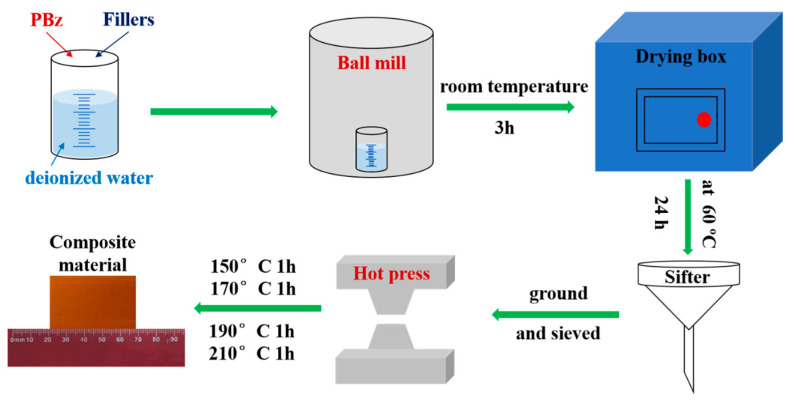
Preparation process of PBz composites.

Figure 2 shows the preparation of PBz Composites. The PBz composites were prepared by a ball-milling-assisted solution mixing method combined with hot pressing. PBz and the fillers (KH560-BN or BN-MgO-SiC_w_) were mixed in deionized water at a designated ratio and ball-milled at room temperature for 3 h. The mixture was then dried at 60 °C for 24 h to obtain mixed powders, which were subsequently ground and sieved to increase fineness. The composites were then fabricated by hot pressing the powders at 12 MPa following a stepwise curing schedule: 150 °C for 1 h, 170 °C for 1 h, 190 °C for 1 h, and 210 °C for 1 h.

## 3. Results and Discussion

### 3.1. Characterization of Modification Results of Fillers

FTIR spectroscopy and Raman spectroscopy were employed to verify the surface modification of BN and SiC_w_ with KH560, as well as the successful synthesis of the BN-MgO-SiC_w_ hybrid filler.

**Figure 3:**
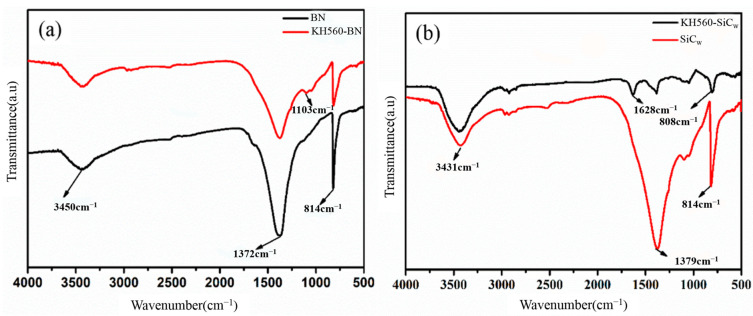

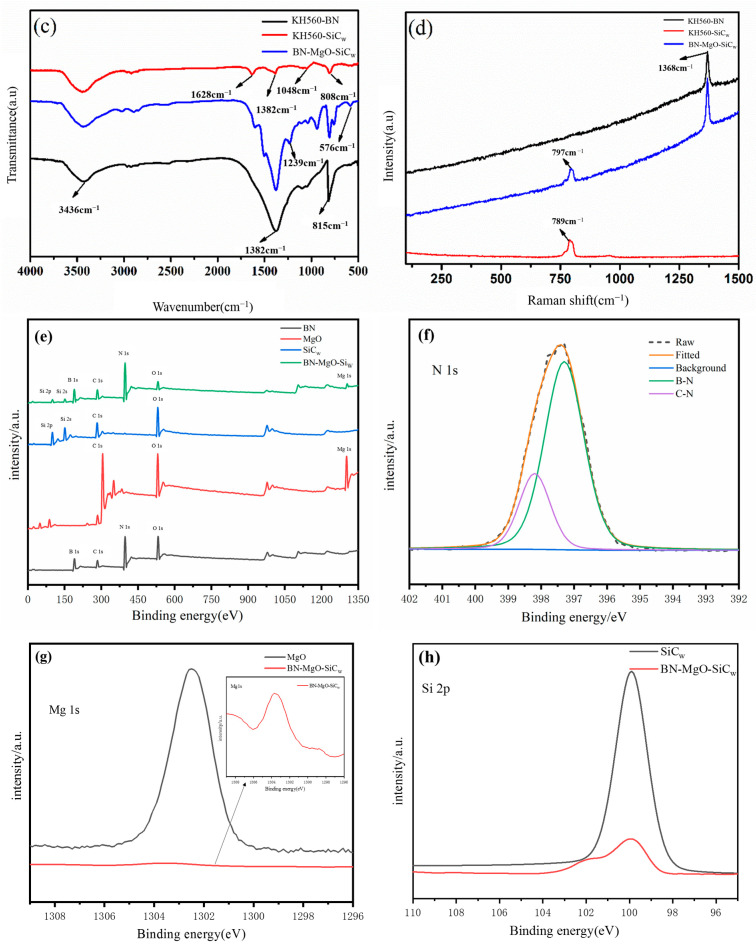

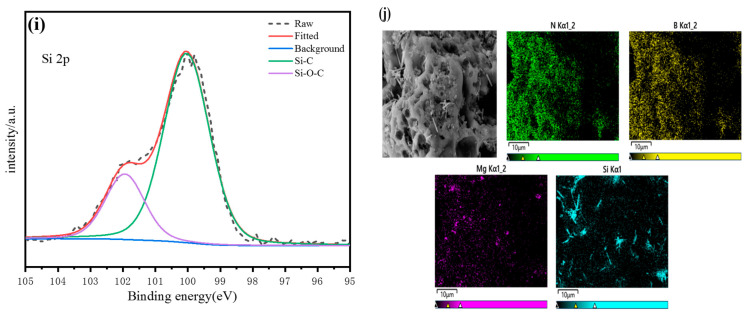
(**a**): FT-IR spectra of BN&KH560-BN; (**b**): FT-IR spectra of SiC_w_&KH560-SiC_w_; (**c**): FT-IR spectra of KH560-BN&KH560-SiC_w_&BN-MgO-SiC_w_; (**d**): Raman spectra of KH560-BN&KH560-SiC_w_&BN-MgO-SiC_w_; (**e**): XPS survey spectra of BN&MgO&SiC_w_ & BN-MgO-SiC_w_; (**f**): N 1s peak spectrum of BN-MgO-SiC_w_; (**g**): MgO & Mg 1s high-resolution spectrum of BN-MgO-SiC_w_; (**h**): Si 2p high-resolution spectrum of SiC_w_& BN-MgO-SiC_w_; (**i**): Si2p peak spectrum of BN-MgO-SiC_w_; (**j**): EDS elemental mapping of BN-MgO-SiC_w_.

Figure 3a presents the FTIR spectra of pristine BN and KH560-BN. The peaks at 1372 cm^−1^ and 814 cm^−1^ correspond to the characteristic absorption bands of B–N bonds, while the peak at 3450 cm^−1^ is attributed to –OH groups. Notably, a new absorption peak appears at approximately 1103 cm^−1^ in the spectrum of KH560-BN, which is characteristic of Si–O bonds. The emergence of this Si–O peak is attributed to the introduction of KH560 molecules, confirming the successful modification of BN.

Figure 3b shows the FTIR spectra of pristine SiC_w_ and KH560-SiC_w_. The characteristic Si–C vibration peak is observed near 814 cm^−1^ in both spectra. Notably, the intensity of the Si–C peak is significantly stronger in pristine SiC_w_ than in KH560-SiC_w_. The peak near 1379 cm^−1^ in both spectra corresponds to C–H bonds. Compared with pristine SiC_w_, the intensity of the C–H peak in the KH560-SiC_w_ spectrum is markedly reduced, indicating successful surface modification of SiC_w_ by KH560.

Figure 3c displays the FTIR spectra of KH560-BN, KH560-SiC_w_, and the BN-MgO-SiC_w_ hybrid filler. Compared with the spectra of KH560-BN and KH560-SiC_w_, two new absorption peaks appear in the BN-MgO-SiC_w_ spectrum at approximately 1239 cm^−1^ and 576 cm^−1^. These peaks correspond to the stretching vibrations of C–N bonds and Mg–O–Si bonds, respectively, which originate from the amide bond and aminosilyl groups. These spectral changes provide strong evidence that BN and SiC_w_ have been successfully covalently linked via NH_2_-MgO.

Figure 3d presents the Raman spectra of KH560-BN, KH560-SiC_w_, and BN-MgO-SiC_w_. The BN-MgO-SiC_w_ hybrid exhibits both the characteristic peaks of KH560-BN and those of KH560-SiC_w_, confirming the integration of both components. The peak near 1368 cm^−1^ in the spectra of BN-MgO-SiC_w_ and KH560-BN is primarily attributed to the E_2_g mode of atomic bond vibrations in KH560-BN. Meanwhile, the peak near 789 cm^−1^ in the spectra of BN-MgO-SiC_w_ and KH560-SiC_w_ corresponds to the E_2_g mode of Si–C bond vibrations. Furthermore, compared with the Raman spectra of KH560-BN and KH560-SiC_w_, the peak positions in the BN-MgO-SiC_w_ spectrum exhibit slight shifts, which are likely due to charge transfer effects. These spectral changes further corroborate the successful covalent linkage between BN and SiC_w_.

To quantitatively investigate the chemical bonding states, XPS survey spectra and high-resolution scans (including N 1s, Mg 1s, and Si 2p) were conducted on the functionalized fillers. As shown in Figure 3e, compared to pristine MgO, the BN-MgO-SiC_w_ hybrid filler exhibits additional N 1s and Si 2p peaks. Quantitative analysis reveals that the surface atomic concentrations of N and Si reach 32.15 at% and 4.11 at%, respectively. These results confirm the successful grafting of the amino-functionalized silane coupling agent (KH540) onto the filler system. High-resolution N 1s spectra of the BN-MgO-SiC_w_ filler were deconvoluted into two distinct components (Figure 3f). The peak located at 397.2 eV corresponds to the B–N bond inherent to BN, while a new characteristic peak appearing at 398.3 eV is attributed to the C–N bond formed during the amidation reaction. Peak area fitting indicates that amino-derived nitrogen accounts for approximately 34% of the total N signal, providing strong evidence that amino groups were retained during the reaction and actively participated in interfacial interactions. Figure 3g presents the Mg 1s spectra of pristine MgO and the BN-MgO-SiC_w_ hybrid filler. The Mg 1s peak for pristine MgO is observed at 1302.5 eV, whereas for the BN-MgO-SiC_w_ filler, it shifts to 1303.5 eV, corresponding to a +1.0 eV chemical shift. This shift is primarily attributed to the formation of Mg–O–Si covalent bonds between MgO and KH540. Due to the higher electronegativity of Si compared to Mg, the electron cloud density around Mg decreases, resulting in an increased binding energy. As shown in Figure 3h, the Si 2p spectrum of pristine SiC_w_ exhibits a symmetric single peak at approximately 100.5 eV, corresponding to Si–C bonds. In contrast, the Si 2p spectrum of the BN-MgO-SiC_w_ filler becomes significantly broader and asymmetric, and can be deconvoluted into two components (Figure 3i). The peak at 100.5 eV is attributed to the Si–C bonds from the SiC_w_ backbone, while the additional peak at 102.1 eV corresponds to Si–O–C bonds, which arise from the interfacial bonding involving KH540. Peak area fitting reveals that a substantial proportion of Si atoms participate in interfacial bonding, providing quantitative evidence for the formation of covalent linkages via the amidation reaction.

EDS elemental mapping was performed on the BN-MgO-SiC_w_ ternary hybrid filler. As shown in Figure 3j, the elemental distribution maps reveal that B (from BN), Si (from SiC_w_), and Mg (from MgO) do not exist as isolated agglomerates. Notably, the Mg signal is spatially located between B and Si, effectively serving as a bridging component. Furthermore, the N signal originates from both BN and the amino groups on the KH540-modified MgO. The EDS maps clearly show that N is uniformly distributed across the Mg-rich interfacial regions, indicating that KH540-MgO indeed functions as a connecting phase. This visual evidence directly supports the formation of a BN-MgO-SiC_w_ ternary filler network.

### 3.2. Microstructure Characterization of Fillers and Composite Materials

**Figure 4:**
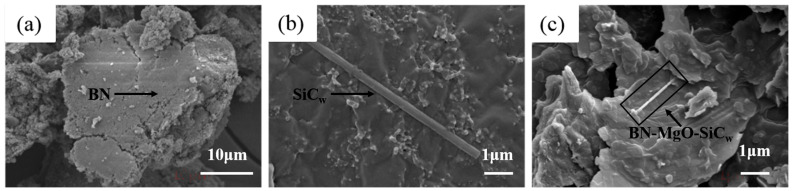
The SEM of fillers: (**a**): m-BN; (**b**):m-SiC_w_; (**c**): BN-MgO-SiC_w_.

Figure 4 shows the microstructures of KH560-BN, KH560-SiC_w_, and the BN-MgO-SiC_w_ hybrid filler. In Figure 4a, the KH560-BN exhibits a sheet-like morphology with a particle size of approximately 24 μm. Figure 4b reveals that KH560-SiC_w_ displays a rod-like structure with a length of approximately 5 μm. Following the amidation reaction, Figure 4c clearly shows that the rod-like SiC_w_ effectively bridges the sheet-like BN particles, forming an interconnected hybrid structure.

**Figure 5:**
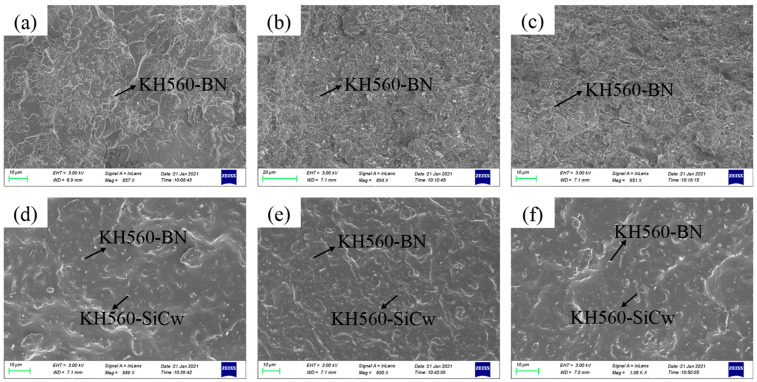
The SEM of composites: (**a**) KH560-BN/PBz-10, (**b**) KH560-BN/PBz-30, (**c**) KH560-BN/PBz-50, (**d**) BN-MgO-SiC_w_/PBz-10, (**e**) BN-MgO-SiC_w_/PBz-30, (**f**) BN-MgO-SiC_w_/PBz-50.

Figure 5 shows the microstructures of KH560-BN/PBz and BN-MgO-SiC_w_/PBz composites at different filler loadings. Figure 5a–c present the microstructures of PBz composites filled with 10 wt%, 30 wt%, and 50 wt% KH560-BN, respectively. Figure 5d–f show the corresponding microstructures of PBz composites filled with 10 wt%, 30 wt%, and 50 wt% BN-MgO-SiC_w_, respectively.

As observed in Figure 5a–c, the distribution of KH560-BN within the polymer matrix becomes increasingly uniform with increasing filler content. At relatively low filler loadings, there is minimal interconnection between adjacent BN sheets. As the filler content increases, both the probability of contact and the contact area between BN particles increase, facilitating the formation of effective thermally conductive pathways within the PBz matrix. This structural evolution promotes heat transfer and consequently enhances the thermal conductivity of the composites.

For the BN-MgO-SiC_w_/PBz composites (Figure 5d–f), a similar trend is observed: the interconnectivity and contact area between fillers increase with higher filler loadings. However, unlike the KH560-BN/PBz system, the BN-MgO-SiC_w_/PBz composites exhibit additional interconnections between the rod-like SiC_w_ and the sheet-like BN particles. This unique morphology contributes to the formation of a more efficient thermal conduction network within the PBz matrix, which is more favorable for heat transfer. Furthermore, the BN-MgO-SiC_w_ hybrid filler demonstrates superior interfacial compatibility with the PBz matrix compared to KH560-BN alone.

As a result, the BN-MgO-SiC_w_/PBz composites exhibit significantly fewer structural defects than their KH560-BN/PBz counterparts. These combined advantages make BN-MgO-SiC_w_ a highly promising filler system for achieving PBz-based composites with excellent thermal conductivity.

To further ascertain the absence of filler agglomeration within the polymer matrix at a loading of 50 wt%, we performed a detailed microzone XRD analysis on the 50 wt% BN-MgO-SiC_w_/PBz composite. Fifteen points on the sample surface were scanned to obtain the integrated intensities (I) of the characteristic diffraction peaks for BN, MgO, and SiC_w_. The intensity ratios, I_BN_/I_MgO_ and I_SiCw_/I_MgO_, were then calculated for each point, as summarized in Table 2. The coefficient of variation (Cv), defined as Cv = (σ/μ) × 100% (where σ is the standard deviation and μ is the mean), was determined to be 10.4% for the I_BN_/I_MgO_ ratio and 12.6% for the I_SiCw_/I_MgO_ ratio. These Cv values, both below the 15% threshold, indicate a uniform distribution of the BN-MgO-SiC_w_ fillers, confirming the absence of significant aggregation or agglomeration.

### 3.3. Thermal Conductivity of Composite Materials

**Figure 6:**
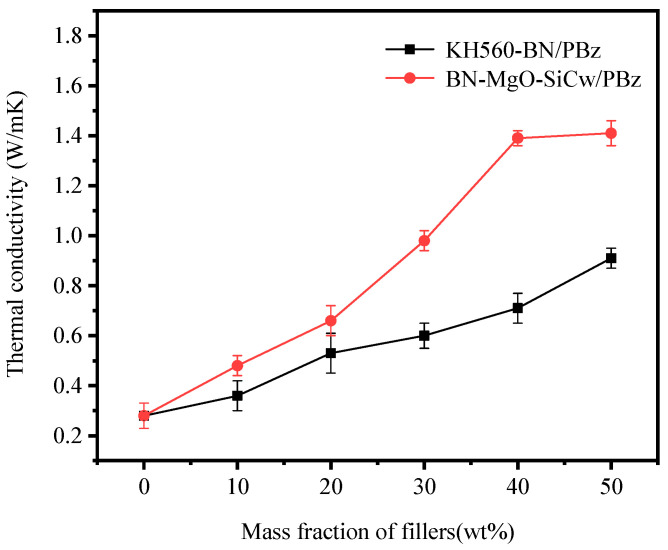
The thermal conductivity of the composite varies with the filler content.

It is well established that the type, content, and thermal conductivity of both the filler and the matrix are key factors influencing the thermal conductivity of polymer composites. Figure 6 presents the thermal conductivity of KH560-BN/PBz and BN-MgO-SiC_w_/PBz composites as a function of filler content. All thermal conductivity measurements were repeated at least five times, and the results are reported as the average values. As clearly shown in the figure, the thermal conductivity of both composite systems increases with increasing filler content. This enhancement can be attributed to the closer packing and progressive alignment of filler particles at higher loadings, which facilitates the formation of more interconnected thermally conductive pathways. Such microstructural evolution effectively shortens the phonon transport distance and accelerates phonon transmission, thereby promoting faster heat diffusion.

**Figure 7:**
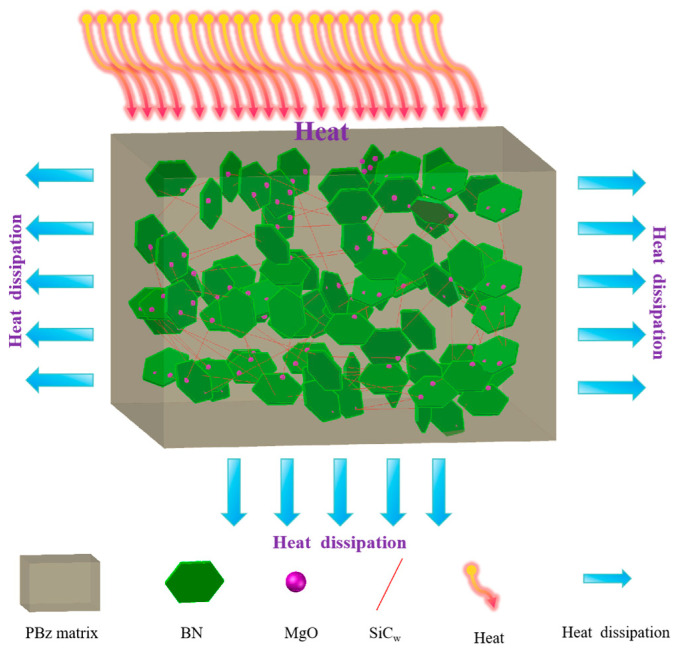
The heat conduction mechanism of BN-MgO-SiC_w_/PBz composite materials.

Furthermore, at equivalent filler loadings, the BN-MgO-SiC_w_/PBz composites exhibit significantly higher thermal conductivity than their KH560-BN/PBz counterparts, with this disparity becoming more pronounced as the filler content increases. At a filler loading of 50 wt%, the thermal conductivity of the BN-MgO-SiC_w_/PBz composite reaches 1.41 W/mK, which is approximately 5.2 times that of the neat PBz matrix. In comparison, the KH560-BN/PBz composite achieves a thermal conductivity of 0.91 W/mK, corresponding to 3.4 times that of the matrix. This enhancement can be attributed primarily to the following two factors. First, the rod-like KH560-SiC_w_ and the sheet-like KH560-BN establish covalent connections through the spherical crosslinking agent KH540-MgO. This configuration enables KH560-SiC_w_ to effectively bridge adjacent KH560-BN particles, facilitating the formation of a more stable and continuous thermally conductive network within the PBz matrix. Such a network accelerates phonon transport and promotes efficient heat diffusion. Second, compared to the binary KH560-BN system, the incorporation of the ternary BN-MgO-SiC_w_ hybrid filler promotes denser packing within the matrix, reducing structural defects and voids. This improved microstructural integrity provides smoother pathways for phonon transport, further enhancing thermal conductivity. Figure 7 schematically illustrates the proposed heat conduction mechanism and corresponding thermal transport model for the BN-MgO-SiC_w_/PBz composite.

### 3.4. Dielectric Properties of Composite Materials

**Figure 8:**
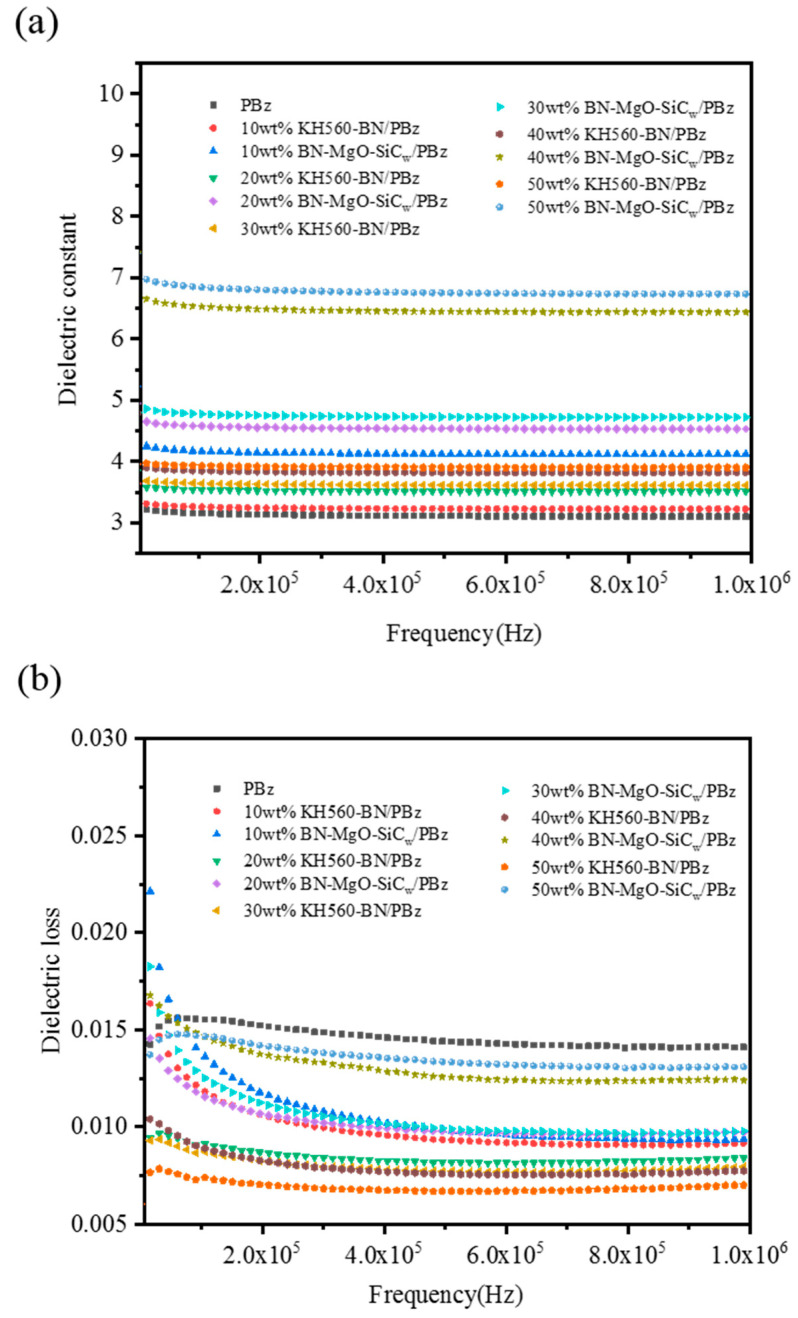
Dielectric properties of composites: (**a**): Dielectric constant; (**b**): Dielectric loss.

In addition to thermal conductivity, the dielectric properties of polymer composites used as microelectronic packaging materials are also very critical. Using polymer compo-sites with lower ε value and tan δ value as electronic packaging materials can significantly reduce the signal attenuation during transmission, thereby improving the working rate of electronic components. Figure 8a characterizes the ε values of BN-MgO-SiC_w_/PBz composites and KH560-BN/PBz composites with different filling content. At a frequency of 1 MHz, the ε value of the neat PBz matrix is 3.07. As the filler content increases, the dielectric constant (ε) of the composites exhibits a nonlinear upward trend. When the loading of KH560-BN reaches 50 wt%, the ε value of the KH560-BN/PBz composite is 3.83. This phenomenon can be theoretically explained using established dielectric models. According to the effective medium theory (EMT), the effective dielectric constant of a two-phase composite is governed by the intrinsic permittivity of the filler, its volume fraction, and the geometric shape factor.

In this study, when the ceramic filler content is below 20 wt%, the Maxwell-Garnett theory applies: the ceramic particles are isolated and dispersed within the polymer matrix, with negligible interaction between fillers. Under these conditions, the increase in the dielectric constant is primarily attributed to the polarization of the high-permittivity ceramic fillers. When the ceramic filler content exceeds 20 wt%, the Bruggeman theory becomes more applicable. Here, the increase in the dielectric constant of the polymer composite arises from two main factors: first, the volume effect resulting from the increased proportion of high-permittivity ceramics; and second, the coupling effect, where interactions between neighboring particles lead to mutual coupling through local electric fields. Even at a ceramic loading of 50 wt%, although the dielectric constant is enhanced compared to the neat matrix and composites with lower filler fractions, it remains below the percolation threshold. Consequently, no sharp increase in ε is observed, allowing the composite to retain favorable dielectric properties. Furthermore, we observed that at the same filler loading, the BN-MgO-SiC_w_/PBz composite exhibits a significantly higher dielectric constant than the KH560-BN/PBz composite. For instance, at a filler content of 50 wt%, the ε value of the BN-MgO-SiC_w_/PBz composite reaches 6.81. This difference can be attributed to the following three factors: 1. Introduction of High-Permittivity Components: SiC_w_ and MgO inherently possess high dielectric constants. Their incorporation into the polymer system directly increases the proportion of high-permittivity components, thereby elevating the overall dielectric constant of the composite. 2. Formation of a Polarization Network through Multi-Dimensional Fillers: The rod-like SiC_w_ whiskers interspersed between the plate-like BN particles create localized polarization micro-regions. This multi-dimensional structure generates numerous enhancement points for the local electric field, leading to a polarization intensity in the composite that exceeds the simple sum of contributions from a single BN filler. 3. Enhanced Interfacial Polarization: The addition of three types of fillers introduces numerous heterogeneous interfaces within the polymer system, including MgO-PBz, SiC_w_-PBz, BN-MgO, BN-SiC_w_, and MgO-SiC_w_. Under an applied electric field, free charges and dipoles are more readily trapped and accumulated at these interfaces. This significant charge accumulation at the interfaces directly contributes to the substantial increase in the dielectric constant of the composite.

Figure 8b shows the dielectric loss (tan δ) of BN-MgO-SiC_w_/PBz and KH560-BN/PBz composites as a function of filler content. As the loading of KH560-BN increases, the tan δ steadily decreases. At a frequency of 1 MHz, the neat PBz matrix exhibits a tan δ of 0.015. When the KH560-BN content reaches 50 wt%, the tan δ of the corresponding composite drops to 0.0071. This reduction can be largely ascribed to the mitigation of interfacial polarization loss resulting from the surface modification of BN with KH560. The treatment promotes the formation of covalent linkages between the BN filler and the PBz matrix, leading to a more uniform and tightly bound interface with fewer micro-voids or structural imperfections. This improved interfacial bonding restricts the mobility of charge carriers, thereby suppressing polarization losses that typically arise from charge accumulation and migration at the interface. Additionally, the oxygen-containing functional groups introduced on the BN surface during modification may trap and immobilize injected charges, further inhibiting carrier transport and contributing to the observed reduction in dielectric loss.

Figure 8b also reveals that, at equivalent filler loadings, the BN-MgO-SiC_w_/PBz composites exhibit higher tan δ values than their KH560-BN/PBz counterparts. For instance, at a filler content of 50 wt%, the tan δ value of the BN-MgO-SiC_w_/PBz composite is 0.013. This phenomenon can be explained from the following perspectives: 1. Formation of Conductive Pathways: The covalent integration of the BN-MgO-SiC_w_ hybrid filler system facilitates the formation of continuous or semi-continuous networks within the PBz matrix. These networks can provide pathways for the long-range transport of charge carriers, allowing charges to migrate over larger distances under an electric field and thus increasing dielectric loss. 2. Intrinsic Carrier Migration in SiC_w_: As a semiconductive material, SiC_w_ contains a substantial number of free charge carriers. Under an external electric field, these carriers may undergo hopping and migration within the established filler network, contributing to the observed energy loss. 3. Enhanced Interfacial Polarization: The significant disparity in dielectric properties between the introduced MgO/SiC_w_ fillers and the PBz matrix leads to substantial charge accumulation at their interfaces. This pronounced interfacial polarization under an applied field directly contributes to an increase in dielectric loss.

It is noteworthy that, despite the higher tanδ values of the BN-MgO-SiC_w_/PBz composites compared to the KH560-BN/PBz system, they remain well within the acceptable range for electronic packaging materials (tanδ < 0.02), satisfying the requirements for most microelectronic packaging applications.

### 3.5. Thermal Stability of Composite Materials

**Figure 9:**
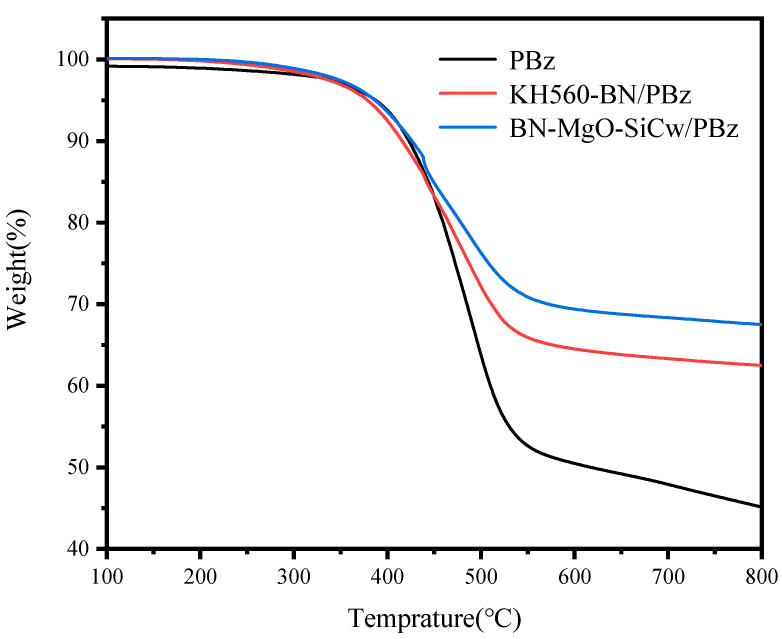
TGA curves of composites.

**Figure 10:**
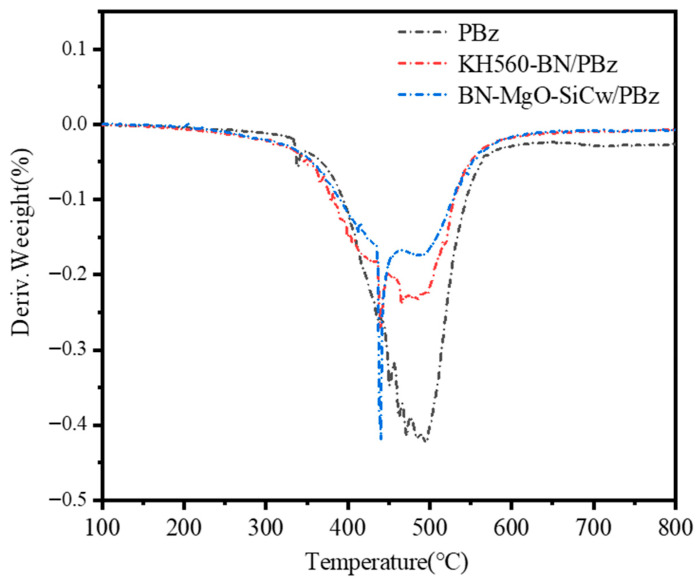
DTG curves of composites.

Figure 9 and Figure 10 present the TGA and DTG curves of the neat PBz matrix, along with the KH560-BN/PBz and BN-MgO-SiC_w_/PBz composites at a filler loading of 50 wt%. The corresponding thermal data are summarized in Table 3. As shown in the TGA curves, the incorporation of thermally conductive fillers significantly increases the char yield at 800 °C for both the KH560-BN/PBz and BN-MgO-SiC_w_/PBz composites compared to the neat PBz matrix.

The DTG curves reveal a sharp, narrow peak at approximately 430 °C for both composite systems, which is absent in the curve of the neat PBz matrix. This peak is attributed to the thermal decomposition of the silane coupling agent anchored on the filler surfaces, which undergoes concentrated degradation around this temperature. While the temperature corresponding to the maximum decomposition rate (T_max_) remains largely similar across all samples, the maximum decomposition rate itself varies considerably. Notably, the BN-MgO-SiC_w_/PBz composite exhibits the lowest maximum decomposition rate, whereas the neat PBz matrix shows the highest, indicating that the hybrid filler system effectively retards the thermal degradation process. For the neat PBz matrix, the thermal degradation temperature corresponding to a 30% weight loss (T_30_) is 485 °C, and the heat resistance index temperature (T_HRI_) is 218 °C. This initial weight loss is primarily attributed to the decomposition of carbonaceous components and the volatilization of polymer fragments. Upon the addition of 50 wt% KH560-BN, the T_30_ and T_HRI_ increase to 511 °C and 224 °C, respectively. More notably, the BN-MgO-SiC_w_/PBz composite with the same filler loading exhibits significantly enhanced thermal stability, with T_30_ and T_HRI_ reaching 572 °C and 244 °C, respectively.

Therefore, we can conclude that due to the interaction between fillers, the thermal degradation of the composite is limited, and both T_30_ and T_HRI_ of BN-MgO-SiC_w_/PBz are significantly increased. Furthermore, under the condition of the same filling content, the improvement degree of BN-MgO-SiC_w_ on T_30_, T_HRI_ and the char yield at 800 °C is significantly greater than that of KH560-BN. This is mainly due to the covalent connection between BN and SiC_w_ in BN-MgO-SiC_w_ and the synergistic effect to further hinder the heat degradation process of the polymer. Moreover, compared with KH560-BN, the BN-MgO-SiC_w_ shows better interfacial compatibility, which is also conducive to the improvement of the thermal stability of PBz composites.

### 3.6. Mechanical Properties of Composite Materials

**Figure 11:**
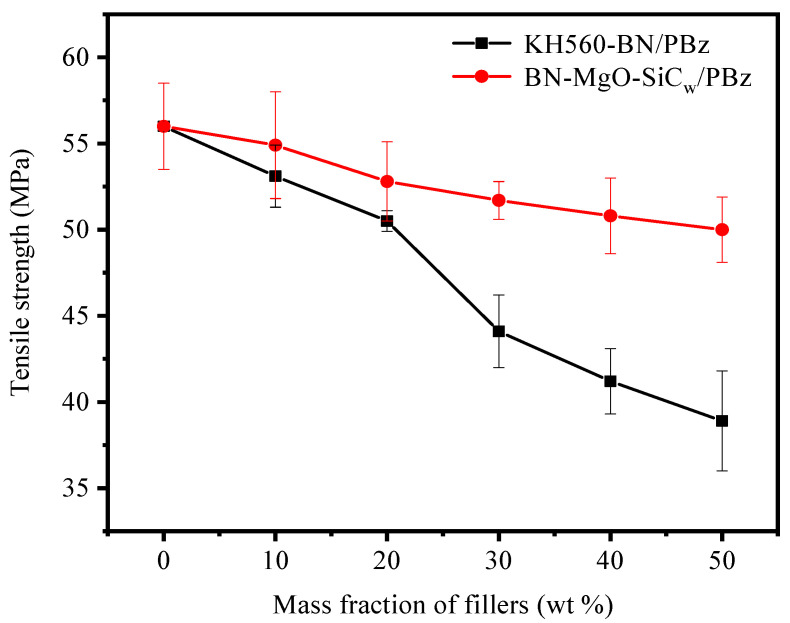
Tensile strength of KH560-BN/PBz and BN-MgO-SiC_w_/PBz.

**Figure 12:**
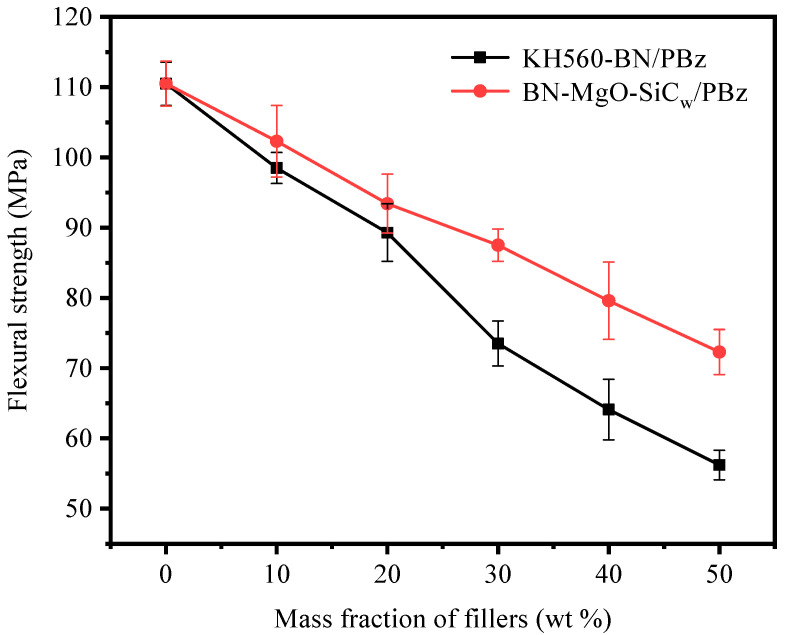
Flexural strength of KH560-BN/PBz and BN-MgO-SiC_w_/PBz.

Figure 11 and Figure 12 illustrate the tensile strength and flexural strength of KH560-BN/PBz and BN-MgO-SiC_w_/PBz composites as a function of filler loading. All tests were repeated at least five times, and the results are reported as the average values. The neat PBz matrix exhibits a tensile strength of 56.0 MPa and a flexural strength of 110.5 MPa. With the incorporation of fillers, both the tensile and flexural strengths of the composites decrease progressively. This decline is primarily attributed to filler agglomeration at higher loadings, which impedes effective stress transfer within the polymer matrix and consequently deteriorates the mechanical properties. Notably, at equivalent filler loadings, the BN-MgO-SiC_w_/PBz composites exhibit significantly higher tensile and flexural strengths compared to their KH560-BN/PBz counterparts. For instance, at a filler content of 50 wt%, the BN-MgO-SiC_w_/PBz composite achieves a tensile strength of 50.0 MPa and a flexural strength of 72.3 MPa, markedly surpassing the values of 39.4 MPa and 56.2 MPa, respectively, observed for KH560-BN/PBz.

This enhancement can be attributed to the following two factors. First, SiC_w_ possesses a high aspect ratio and excellent intrinsic mechanical properties, enabling it to function as an effective reinforcing agent within the polymer matrix. Second, the covalent linkages formed between KH560-modified BN and KH560-modified SiC_w_ promote a stable synergistic interaction, further improving the load-bearing capacity and tensile performance of the BN-MgO-SiC_w_/PBz composites.

**Figure 13:**
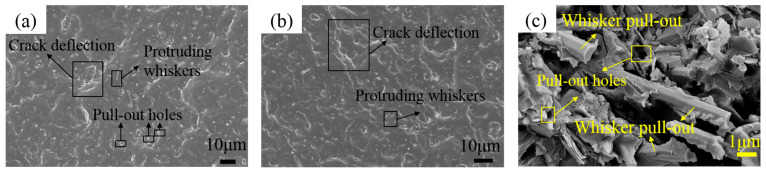
Fracture morphology of BN-MgO-SiC_w_/PBz: (**a**,**b**): Overall fracture morphology; (**c**) local magnified fracture morphology.

Analysis of the tensile fracture surfaces via SEM provides further insight into the mechanical behavior of the composites. As shown in Figure 13a,b, the relatively rough fracture surfaces with visible whisker protrusions suggest a toughened brittle fracture process. In Figure 13c, two distinct features can be observed: voids left by whiskers pulled out from the matrix and fractured SiC_w_ whiskers. The presence of residual resin adhered to the surface of the pulled-out whiskers indicates strong interfacial adhesion between the SiC_w_ and the PBz matrix. Meanwhile, the fractured whiskers confirm that the SiC_w_ effectively bore and transferred stress during loading, providing direct evidence of the typical whisker pull-out and bridging mechanisms.

Furthermore, as illustrated in Figure 13a,b, crack propagation appears to have undergone significant longitudinal deflection rather than continuing along the original fracture plane. This deviation occurs because, upon encountering SiC_w_ whiskers, the advancing crack does not immediately propagate through them; instead, the whiskers are initially pulled out from the matrix. This pull-out process consumes additional fracture energy, thereby inducing crack deflection and enhancing the overall toughness of the composite.

In comparison to the KH560-BN/PBz composites, the BN-MgO-SiC_w_/PBz system exhibits notably improved tensile and flexural strengths. These enhancements substantiate the reinforcing role of SiC_w_ within the polymer matrix. The high aspect ratio and outstanding mechanical properties of SiC_w_ enable effective energy dissipation through whisker bridging and crack deflection mechanisms, which collectively contribute to the improved mechanical performance of the composites [39].

## 4. Conclusions

In summary, BN-MgO-SiC_w_/PBz composites were successfully fabricated via ball-milling-assisted solution mixing followed by hot-press molding. In this approach, KH540-functionalized MgO served as a crosslinking agent, enabling covalent linkage between KH560-BN and KH560-SiC_w_ through an amidation reaction.

The BN-MgO-SiC_w_ hybrid filler was incorporated into the PBz matrix to enhance the thermal conductivity, thermal stability, dielectric properties, and mechanical performance of the resulting composites. Owing to the covalent bonding between BN and SiC_w_ and the resulting synergistic effect, the composite with 50 wt% BN-MgO-SiC_w_ achieved a thermal conductivity of 1.41 W/mK, which is approximately 5.8 times that of the neat PBz matrix. Due to the intrinsically high dielectric constant and dielectric loss of SiC_w_, the dielectric performance of the BN-MgO-SiC_w_/PBz composite is slightly inferior to that of the KH560-BN/PBz composite at equivalent filler loadings.

The BN-MgO-SiC_w_/PBz composite also exhibits excellent thermal stability, attributed to the interactions between the filler components. The thermal degradation temperature at 30% weight loss (T_30_) and the heat resistance index temperature (T_HRI_) reach 572 °C and 244 °C, respectively, both significantly higher than those of the neat PBz matrix.

With increasing thermally conductive filler content, the tensile strength and flexural strength of the composites progressively decrease. However, compared with the KH560-BN/PBz system, the incorporation of BN-MgO-SiC_w_ substantially improves the mechanical properties. At a filler loading of 50 wt%, the BN-MgO-SiC_w_/PBz composite exhibits a tensile strength of 50.0 MPa and a flexural strength of 72.3 MPa. This enhancement is primarily attributed to the high aspect ratio of SiC_w_, as well as the synergistic interactions between BN and SiC_w_ within the hybrid filler system.

## Figures and Tables

**Table 1:** Performance comparison of composite materials from different systems.

Material Type	Thermal Conductivity (W/mk)	Dielectric Constant	Dielectric Loss	Tg (°C)	Tensile Strength (Mpa)
A-ph/CE/h-BN@PDA [33]	0.71	5.72 (1 KHz)	0.0148 (1 KHz)	339.9	-
(m/n)BN/PBz [34]	0.9196	3.45 (1 MHz)	0.005 (1 MHz)	216	-
SiC@SiO_2_ whiskers/PI [35]	0.95	16.2 (1 MHz)	0.44 (1 MHz)	378	48.97
EP/SiO_2_@BN [36]	0.86	-	-	-	-
PI/AlN/BN [37]	0.711	7.9 (1 MHz)	0.035 (1 MHz)	-	100
BN@15CNT/PBz [38]	0.794	1.6 (1 MHz)	0.028 (1 MHz)	218	-
BN-MgO-SiC_w_/PBz	1.41	6.81 (1 MHz)	0.013 (1 MHz)	244	49.97

**Table 2:** The integrated intensities.

Number	I_BN_/I_MgO_	I_SiCw_/I_MgO_	Number	I_BN_/I_MgO_	I_SiC_/I_MgO_
1	39.8	11.7	9	45.5	10.7
2	46.6	9.3	10	36.5	9.1
3	34.2	8.5	11	40.1	8.1
4	32.9	7.1	12	38.4	8.3
5	44.7	8.9	13	44.9	11.0
6	35.9	8.8	14	42.2	10.5
7	39.1	9.1	15	36.2	9.8
8	41.3	9.2	--	--	--
μ	39.9	9.34	--	--	--
σ	4.14	1.17	--	--	--
Cv	10.4%	12.5%	--	--	--

**Table 3:** Thermal date of composites.

Sample	TG (°C)		T_HRI_ a/°C	Char Yield @800 °C %
	5%/wt	30%/wt		
PBz	387	485	218	45.0
KH560-BN/PBz	379	511	224	62.6
BN-MgO-SiC_w_/PBz	386	572	244	67.5

T heat-resistance index a/°C = 0.49 × [T_5_ + 0.6 × (T_30_ − T_5_)].

## Data Availability

The original contributions presented in this study are included in the article. Further inquiries can be directed to the corresponding author.

## Abbreviations

The following abbreviations are used in this manuscript:

BN	boron nitride
SiC_w_	silicon carbide whisker
PBz	polybenzoxazine
PF	phenol-formaldehyde resin
PI	polyimide
EP	epoxy resin
BMI	bismaleimide resin
THF	tetrahydrofuran
